# Effect of the WHO ICOPE prevention program on falls and mobility functions : a retrospective study in France

**DOI:** 10.1186/s12877-026-08073-7

**Published:** 2026-08-11

**Authors:** Pierrot Boishus, Caroline Berbon, Néda Tavassoli, Justine De Kerimel, Laure Bouchon, Florence Da Costa Ribeiro, Magali Poly, Charlene Seguela, Sandrine Augusto, Catherine Takeda, Maria-Eugenia Soto-Martin

**Affiliations:** 1 IHU HealthAge & WHO Collaborating Center for Frailty, Clinical & Geroscience Research, and Geriatric Training, Toulouse University Hospital, Toulouse, France; 2 Aging Research Team, CERPOP, INSERM UMR1295, University of Toulouse, Toulouse, France

**Keywords:** Older adults, Fall, ICOPE program, Intrinsic capacity, Multifactorial intervention

## Abstract

**Background:**

World Health Organization developed the Integrated Care for Older People (ICOPE) prevention program in order to promote the maintenance of intrinsic capacities for older adults, based on six key domains. Each key domain is a risk factor for falls in older adults, they are accessible to interventions. The ICOPE prevention program involves screening test, in-depth assessment and development of an interventional personalized care plan for the participant. As the aim of the ICOPE program, fall risk evaluation and management must be multidomain and personalized. The objective of this study was to evaluate the association between the completion of an in-depth assessment followed by a personalized care plan, and the occurrence of falls in older adults, within the approximate interval of one year.

**Method:**

This is an observational retrospective cohort study including 357 participants, as part of the Toulouse Gérontopôle’s frailty prevention and management actions (France). They all had a personalized care plan following an initial in-depth assessment, and a second in-depth assessment conducted approximately one year later. During these assessments, data were collected during an in-person individual evaluation conducted by a nurse, using tools as questionnaires, scales, stopwatches, measuring tapes and handgrip dynamometers. We collected data on self-reported falls within the three months preceding the assessments, and clinical mobility-related elements such as the Short Physical Performance Battery (SPPB), self-reported concerns about falling, and frailty criteria according to Fried’s score including gait speed. We compared the initial status between self-reported fallers in the last 3 months versus non-fallers, using a multivariate logistic regression model to examine the factors associated with a history of falls at the initial assessment. We compared the proportion of self-reported fallers and participant’s mobility-related elements approximately one year after the personalized care plan was proposed, using McNemar test, Student’s T-test or the Wilcoxon rank-sum test, depending on the variables. We compared the participants’ results based on their level of adherence to the care plan, using the Chi-square test or Fisher’s exact test for categorical variables, for continuous variables analysis of variance (ANOVA) or the Kruskal-Wallis test was used.

**Results:**

Approximately one year after the first in-depth assessment, there were not statistically significant difference in the occurrence of falls in the last three months (20.5% reported a fall in the past three months at the first in-depth assessment versus 15.7% at the second in-depth assessment, *p* = 0.07). There were significantly different results based on the level of care plan adherence regarding the SPPB score (median 11 (8–12), 10 (7–12) and 11 (9–12) ; respectively for care plan not implemented, partially implemented and fully implemented *p* = 0.008), gait speed expressed in time to walk 4 m in seconds (mean 4.8 +/- 2.3, 5.3 +/- 2.2 and 4.6 +/-1.5 ; respectively for care plan not implemented, partially implemented and fully implemented, *p* < 0.01), Fried score (median 2 (1–2), 1 (0.5-3) and 1 (0–2) ; respectively for care plan not implemented, partially implemented and fully implemented, *p* = 0.001), and concerns about falling (26.8%, 44.4% and 27.4% ; respectively for care plan not implemented, partially implemented and fully implemented, *p* = 0.003). The group that partially implemented the care plan seems to stand out negatively in comparison to both not implemented and fully regarding the SPPB score, gait speed, Fried status and concerns about falling.

**Conclusion:**

Subgroup analyses revealed significant differences one year after the submission of the care plan concerning mobility parameters based on the level of plan implementation, highlighting the importance of this parameter. A secondary analysis suggests that those who were initially more robust (with a lower Fried frailty score) appear to have fully adhered to the recommended measures.

**Supplementary Information:**

The online version contains supplementary material available at https://doi.org/10.1186/s12877-026-08073-7.

## Background

For ethical, economic, and societal reasons, worldwide demographic transition underscores the need to orient healthcare systems towards the goal of healthy aging, as defined by the World Health Organization (WHO) as the process of developing and maintaining the functional ability that enables well-being in older age [1]. Functional ability is composed of Intrinsic Capacity (IC) (representing the composite of all the physical and mental functions of an individual), of environment characteristics, and the interaction between them. IC is based on six domains : mobility, nutrition, cognition, psychology, hearing and vision. During life, IC tends to decline due to a decrease in the individual’s functional reserve, requiring a multidimensional approach to promote its maintenance [1]. In this context, WHO develop the Integrated Care for Older People (ICOPE) prevention program, with the objective to decelerate the decline of IC, stabilize it, or even reverse its trend [2].

Decline in IC is a risk factor for geriatric syndromes such as falls, which are a major public health issue for older adults [1]. Between 28% and 35% of community dwelling older adults aged more than 65 experiment a fall each year globally [3]. In France, this same proportion is found where approximately one third of 65-year-old people, and a half of 85-year-old people fall each year [4]. Falls can lead to injuries, negative psychological impact, higher use of emergency care and hospitalizations, loss of functional independence, and death [5]. In France, its economic consequences are estimated to be 2 billion euros each year [6].

Each IC key domain represents a fall risk factor in old people. ICOPE Step 1 aims to detect a potential decline in each IC key domain with fast and easy screening tests. Those domains are accessible to interventions. Step 2 of ICOPE prevention program is an in-depth assessment of the key domains in order to build an interventional personalized care plan (Step 3) based on the assessment and priorities of the individual [7]. Fall risk evaluation and management must be multidomain and personalized [2], as the aim of the ICOPE prevention program.

Our hypothesis was that performing an ICOPE Step 2 in-depth assessment followed by the achievement of the personalized care plan (Step 3) could have a beneficial effect on the occurrence of falls.

The objective of this study was to evaluate the association between the completion of an in-depth assessment followed by a personalized care plan, and the occurrence of falls, within the approximate interval of one year.

In detail, we 1) compared the initial status between fallers versus non-fallers, 2) compared the occurrence of falls in the last three months and mobility-related elements approximately one year after the personalized care plan was proposed, and 3) compared the participants’ results based on their level of adherence to the care plan.

## Method

**Fig. 1:**
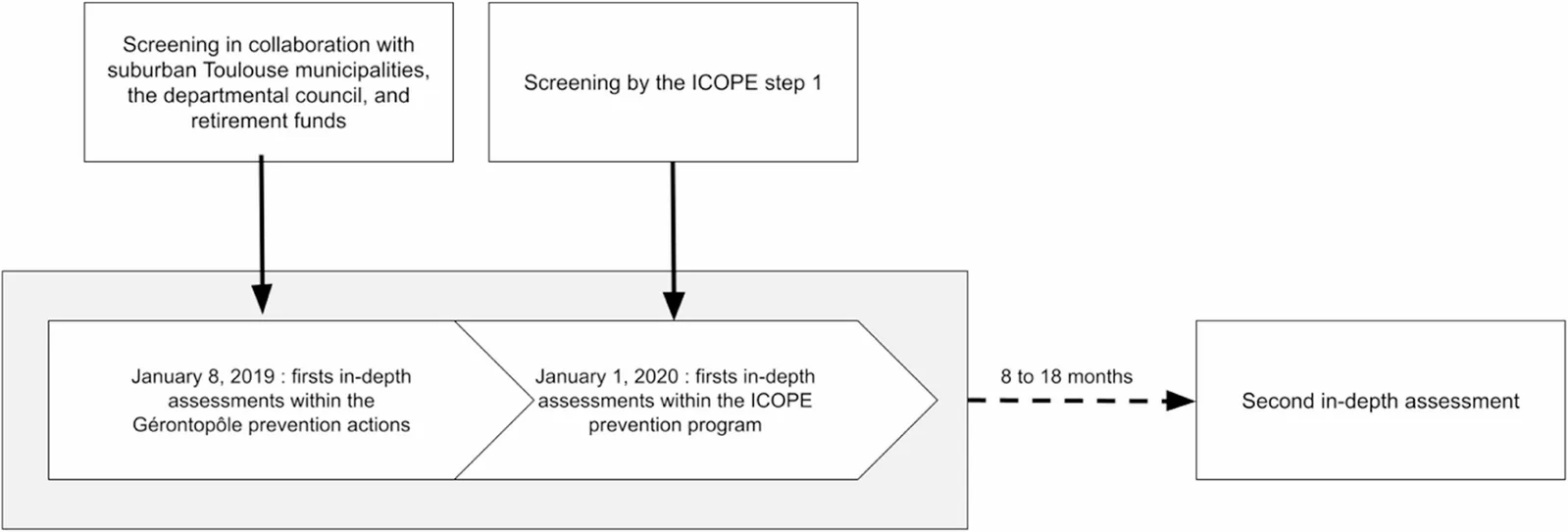
Enrollment of the population in the prevention pathway, initially through the Gerontopole’s prevention actions, and then through the ICOPE program

This is an observational retrospective cohort study. Participants were recruited through multiple pathways as detailed below. Starting on January 2019, participants were included as part of the Toulouse Gérontopôle’s frailty prevention and management actions, in collaboration with suburban Toulouse municipalities, the departmental council and retirement funds. A self-screening frailty questionnaire was previously sent to older adults which agreed to be recontact by a Gérontopôle’s nurse. If the results of this self-assessment indicated a need, the nurse would then offer the senior an in-depth assessment. Starting from January 1th, 2020, these assessments were conducted as part of the ICOPE program. Older adults were included by the Step 1 ICOPE screening test (conducted as a self-assessment, by a healthcare professional (HCP), by a caregiver, or by a non-HCP). If necessary, according to the Step 1 results, a Gérontopôle’s nurse proposed a Step 2 in-depth assessment. All the participants were offered a personalized care plan following the in-depth assessment, regardless of the inclusion method. One year after the first in-depth assessment, a second Step 2 was conducted, gathering the same evaluation data and including a question about the implementation of the care plan and recommendations. Figure 1 illustrates the participants’ inclusion method.

### Studied population

To be included in this study, participants had to be aged 60 years and older, be independent in activities of daily living (Katz ADL ≥ 5), and have completed at least 2 in-depth assessments approximately 1 year apart (an interval of 8 to 18 months was selected to correspond with real-world clinical practice). Additionally, complete data from these evaluations were required regarding : the occurrence of a fall in the last 3 months ; their score on the Short Physical Performance Battery (SPPB) [8], including walking speed ; the presence or absence of concerns about falling and the presence or absence of frailty criteria according to Fried [9], and the number of such criteria.

For the second in-depth assessment, data regarding the level of recommendations implementation performed following the first Step 2 in-depth assessment was also required (full, partial, or lack of implementation).

### Data collection

Data came from the ICOPE MONITOR Database of Toulouse University Hospital. The data were collected during an in-person individual interview conducted by a registered nurse trained in performing in-depth assessment. The registered nurses underwent a two-day training session conducted by instructors from the Toulouse University Hospital. The training program addresses the use and interpretation of standardized assessment scales, the integration of individual life goals and environmental factors, and the development of a personalized care plan. The educational approach combines theoretical instruction, practical exercises, and analysis of clinical case studies. An optional one-day practical session can complement the training. The in-depth assessment explores the six IC domains of the WHO’s ICOPE program through detailed tests and questionnaires, including the SPPB test, the self-reported occurrence of a fall in the last 3 months, the self-reported concerns about falling (by asking participants “Do you experience concerns about falling?”, with a binary response option : “yes” or “no”), and the frailty criteria according to Fried’s score. At the end of the in-depth assessment, a care plan was proposed, including health interventions that may benefit the participant based on the results observed in the six domains and the individual’s preferences (Step 3). This plan was documented in the database. The care plan might include, for example, referral to specialized consultations, advice on environmental modifications, nutritional guidance including the prescription of oral nutritional supplements, recommendations for physical exercise either independently or under supervision, and referrals to associations. Subsequently, the participant was invited for another in-depth evaluation one year after the first one. During the second in-depth assessment, the adherence to recommendations was assessed by the question, “Have the recommendations from the previous visit have been implemented?” with a binary response option (“Yes” or “No”). If “Yes,” it was then asked whether they were followed “Fully” or “Partially”.

### Ethical considerations

This study was conducted according to the guidelines of the Declaration of Helsinki. ICOPE MONITOR database complies with data confidentiality and security requirements according to the general data protection regulation and was validated by the National Commission on Informatics and Liberty (CNIL) in 2017 (registration number 247169284s, reference MMS/OSS/NDT171027). All participant enrolled in the prevention program were presented with an information notice explicitly informing them that Toulouse University Hospital may access and reuse their data for the establishment of a database dedicated for future scientific research projects, within the framework of the INSPIRE - ICOPE CARE Program proposed by Toulouse’s Gerontopole. Participants were also informed of their right to withdraw and were given the relevant contact. This MR-004 study is registered with the Research and Innovation Department of Toulouse University Hospital under reference RnIPH 2023 − 122.

### Studied variables

The population was described based on the results of the in-depth assessments : history of falls in the last 3 months, age, gender, number of comorbidities and medications, lifestyle, presence of home assistance, were self-reported data. Mini Mental State Examination (MMSE) was administered in its validated French Version, in accordance with the recommendations of the French National Authority for Health (HAS), to evaluate cognitive functioning in community dwelling older adults [10–12]. An unauthorized version of the French MMSE was used by the study team without permission, however this has now been rectified with PAR. The MMSE is a copyrighted instrument and may not be used or reproduced in whole or in part, in any form or language, or by any means without written permission of PAR (www.parinc.com). Higher score indicates better cognition with a maximum score of 30. In the meta-analysis, the fixed-effects pooled proportion for sensitivity of the MMSE was 77% [13]. Its reliability is estimated to be 0.75 [14]. The SPPB [8] is a composite score ranging from 0 to 12 points, where 4 points can be assigned based on walking speed, 4 points based on the time required to complete 5 chair stands, and 4 points based on the performance of balance tests in standing with feet together, in semi-tandem, and in tandem. Low performance is considered for scores ranging from 0 to 6, intermediate performance for scores from 7 to 9, and high performance for scores of 10 or above. The SPPB score demonstrated an intra-rater reliability of 0.88 and an inter-rater reliability of 0.86 [15].

The duration of unipedal stance (measured) is considered abnormal if it is less than 5 s [16, 17]. Concerns about falling is a self-reported data. The Fried frailty score [9] is a composite score where one point is assigned for each of the following criteria : unintentional weight loss of 4.5 kg or more in one year (self-reported), self-reported exhaustion, measured grip strength below reference values, walking speed less than 0.8 m/s (measured), and low physical activity (self-reported). A participant was considered robust (non-frail) with a score of 0, pre-frail with a score of 1 or 2, and frail with a score of 3 or more out of 5. Frailty phenotype is reproducible and has been shown to identify individuals at elevated risk for disability, institutionalization, hospitalization, and mortality [9, 18, 19]. Instrumental Activities of Daily Living (IADL) [20] was a self-reported data. Lawton IADL scale showed very good intra-rater and inter-rater reliability [21, 22]. Mini Nutritional Assessment (MNA) [23] was administered in its French version. Nutritional status according to the MNA was considered normal for a score of 24 points or higher, at risk of malnutrition for a score between 17 and 23.5 points, and poor nutritional status for a score below 17 points. MNA is a validated tool for detecting malnutrition and risk of malnutrition for older adults, with a sensitivity of 80% [24]. Body Mass Index (BMI) was expressed in kg/m², with height being self-reported and weight measured during the evaluation. The 4-meter walking time was expressed in seconds. The level of adherence to the care plan was a self-reported data (fully, partially, or not implemented) collected during the second in-depth assessment.

### Statistical analysis

Categorical variables are presented by their frequencies (N) and percentages (%). Continuous variables are described by their mean and standard deviation or by the median and interquartile range, depending on their distribution. The comparison of variables between fallers and non-fallers was performed using the Chi-square test or Fisher’s exact test for frequency comparisons. Continuous variables were compared using the Student’s T-test or the Mann-Whitney test. A multivariate logistic regression model was used to examine the factors associated with a history of falls at the initial assessment. A stepwise regression was conducted with the variables of interest retained at a threshold of 0.20 in the bivariate analyses. A comparison of results between the initial assessment and the follow-up assessment was performed. The tests used to compare these results were the McNemar test for frequency comparisons. Continuous variables were compared using the Student’s T-test or the Wilcoxon rank-sum test, depending on the distribution of the variables. The comparison of results at follow-up step 2 based on the level of adherence to the care plan (not at all, partially, or fully) was conducted using the Chi-square test or Fisher’s exact test for categorical variables. For continuous variables, analysis of variance (ANOVA) or the Kruskal-Wallis test was used. The analyses for this study were conducted using the Stata software package (StataCorp LP, College Station, TX, USA), version 14.2.

## Results

**Fig. 2:**
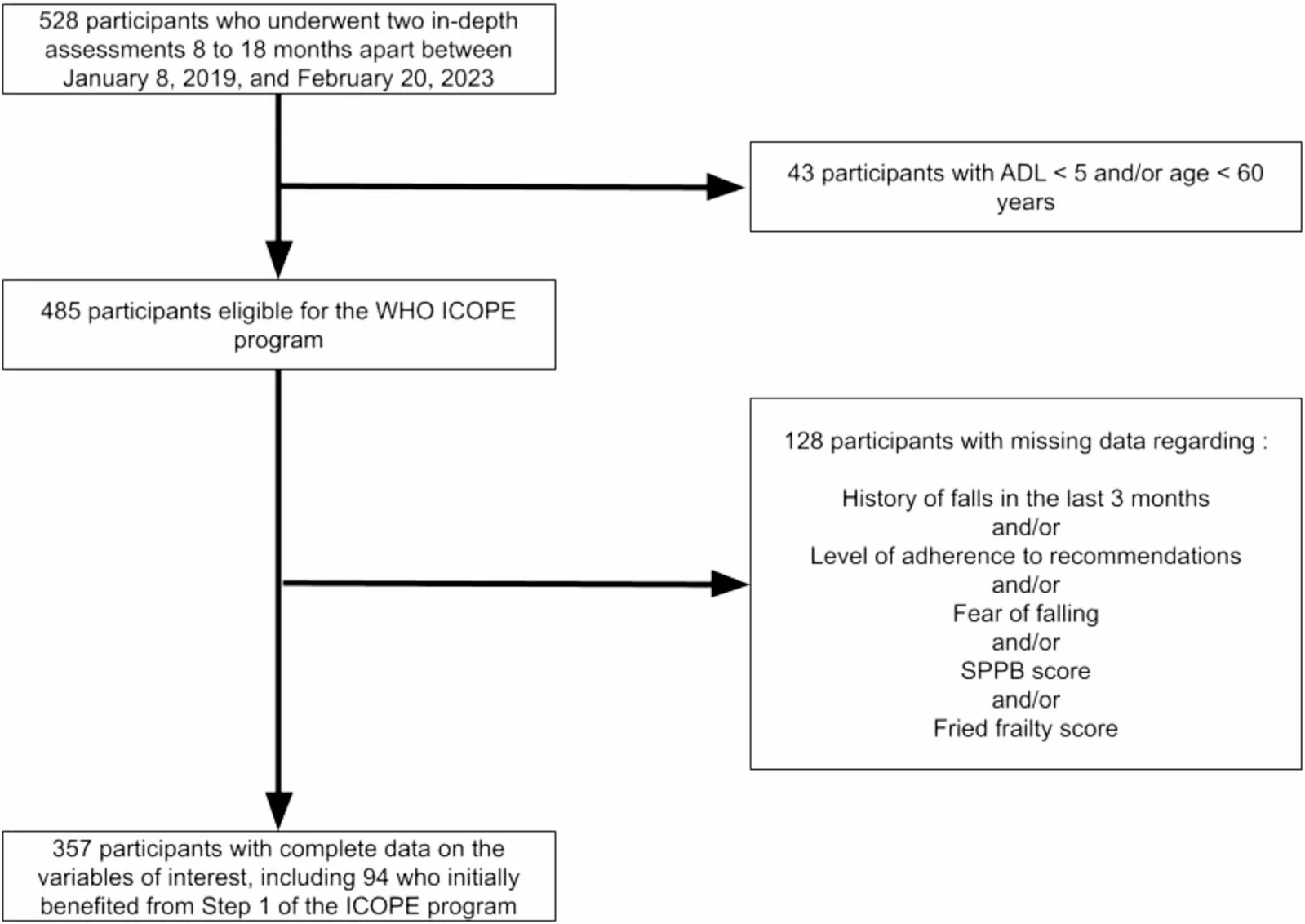
Inclusion of the study population

Between January 8, 2019, and February 20, 2023, 528 participants underwent two in-depth assessments conducted approximately one year apart (8–18 months). Among this sample, 43 participants did not meet the inclusion criteria, having either an ADL score below 5 or an age below 60 years. Among the 485 ICOPE-eligible participants, 357 presented complete data for all variables of interest and were included (see the flowchart in Fig. 2). Participants’ characteristics at the initial in-depth assessment can be seen in supplementary data, their mean age is 77.8 years (±7.3). The comparison between participants reporting a fall (*N* = 73) and those not reporting one (*N* = 284) during the initial in-depth assessment in bivariate analysis is shown in Table 1. In the multivariate model, factors associated with a history of falls included older age (OR 1.09; *p* = 0.003), with female gender approaching significance (OR 2.4; *p* = 0.05), and an increased MNA appears as a protective factor (OR 0.85; *p* = 0.007).

**Table 1:** Comparison of fallers and non-fallers during the initial in-depth assessment, bivariate analysis

			Fallers (*N* = 73)	Non-fallers (*N* = 284)	*p*
Age*, in year			**81.0+/-7.2**	**77.0+/-7.1**	**< 0.01**
Gender, female			**51 (69.9)**	**180 (63.4)**	**< 0.01**
Number of comorbidities**			**3 (2–3)**	**2 (1–3)**	**0.04**
Number of medications*			**5.2+/-2.8**	**4.0+/-3.2**	**0.007**
Living habits, alone			39 (53.4)	126 (44.4)	0.17
Home care assistance, yes			**43 (58.9)**	**114 (40.1)**	**0.004**
Cognition	MMSE*		25.7+/-3.1	26.5+/-3.2	0.09
Mobility	SPPB**		**8 (6–10)**	**11 (9–12)**	**< 0.01**
	Performance on the SPPB	High	**29 (39.7)**	**194 (68.3)**	**< 0.01**
		Intermediate	**21 (28.8)**	**58 (20.4)**	
		Low	**23 (31.5)**	**32 (11.3)**	
	Unipedal stance, normal		32 (43.8)	163 (57.4)	0.09
	Concerns about falling, yes		**53 (72.6)**	**81 (28.5)**	**< 0.01**
	Fried score **		**2 (1–3)**	**1 (0–2)**	**< 0.01**
	Fried status	Non-frail	**10 (13.7)**	**87 (30.6)**	**< 0.01**
		Pre-frail	**34 (46.6))**	**148 (52.1)**	
		Frail	**29 (39.7)**	**49 (17.3)**	
	IADL**		**8 (6–8)**	**8 (7–8)**	**0.002**
Nutrition	MNA*		**24.4+/-3.5**	**26.3+/-2.7**	**< 0.01**
	Nutritional status according to MNA	Normal	**43 (58.9)**	**207 (72.9)**	**0.03**
		At risk	**14 (19.2)**	**39 (13.7)**	
		Malnutrition	**2 (2.7)**	**1 (0.4)**	
		MD	**14 (19.2)**	**37 (13.0)**	
	BMI in kg/m²*		25.5+/-4.3	26.5+/-4.7	0.1

*MMSE* Mini-Mental State Examination (from 0 to 30), *SPPB* Short Physical Performance Battery (from 0 to 12), *IADL* Instrumental Activity of Daily Living Score of Lawton (from 0 to 8), *MNA* Mini Nutritional Assessment (from 0 to 30), *BMI* Body Mass Index, *MD* Missing Data

The data are presented as *mean ± SD or **median (p25-p75) or *n* (%)

Bold = *p*<0.05

At the second in-depth assessment, 15.7% of participants reported a fall in the past three months compared to 20.5% at the first in-depth assessment, which did not represent a statistically significant difference (*p* = 0.07). Performance on the SPPB, the Fried score, and concerns about falling were not significantly different between the first and second in-depth assessment. Walking speed, measured by the time to walk 4 m, was significantly slower at the second in-depth assessment (mean time of 4.8 ± 1.7 s at the first assessment versus 5.0 ± 2.07 s at the second assessment, *p* = 0.03). See Table 2.

**Table 2:** Comparison of fall history, mobility and frailty parameters at one-year interval

		Initial assessment (*N* = 357)	Follow-up assessment (*N* = 357)	*p*
Fall history in the past 3 months, yes		73 (20.5)	56 (15.7)	0.07
Total SPPB score **		11 (8–12)	11 (8–12)	0.3
Time to walk 4 m in seconds*		**4.8+/-1.7**	**5.0+/-2.07**	**0.03**
Performance on the SPPB	High	223 (62.5)	225 (63.0)	0.9
	Intermediate	55 (15.4)	58 (16.3)	
	Low	79 (22.1)	74 (20.7)	
Fried score **		1 (0–2)	1 (0–2)	0.9
Fried status	Non-frail	97 (27.2))	105 (29.4)	0.4
	Pre-frail	182 (51.0)	172 (48.2)	
	Frail	78 (21.8)	80 (22.4)	
Concerns about falling, yes		134 (37.6)	128 (35.9)	0.6

The data are presented as *n* (%) or *mean ± SD or **median (p25-p75)

Bold = *p*<0.05

Table 3 compares, at the follow-up assessment, fall history, mobility and frailty parameters according to the level of adherence to the care plan (fully implemented, partially implemented, not implemented). There were no statistically significant differences in fall history in the past three months between the three groups. However, there were significantly different results regarding the SPPB score, walking speed, Fried status, and concerns about falling based on the level of care plan adherence, with the group that partially implemented the care plan exhibiting lower performance than the other two groups : their median SPPB score is 10 versus 11 in the other two groups ; their mean time to walk 4 m in seconds is 5.3 +/- 2.2 versus 4.8 +/- 2.3 for the group not implemented and 4.6 +/- 1.5 for the group fully implemented ; their percentage of frail participants is higher than in the other groups (26.1% versus 23.2% for the group not implemented and 14.7% for the group fully implemented) ; 44.4% of participants in group partially implemented report concerns about falling, versus 26.8% for the group not implemented and 27.4% for the group fully implemented. We note that the fully implemented group shows a higher proportion of non-frail participants compared to the other two groups (45.3% versus 27% for partially implemented group, and 20.7% for not implemented group).

**Table 3:** Comparison of fall history, mobility and frailty parameters at follow-up assessment according to the level of care plan adherence

		Care plan not implemented (*N* = 82)	Care plan partially implemented (*N* = 180)	Care plan fully implemented (*N* = 95)	*p*
Fall history in the past 3 months, yes		12 (14.6)	33 (18.3)	11 (11.6)	0.3
Total SPPB score**		**11 (8–12)**	**10 (7–12)**	**11 (9–12)**	**0.008**
Time to walk 4 m in seconds*		**4.8+/-2.3**	**5.3+/-2.2**	**4.6+/-1.5**	**< 0.01**
Performance on the SPPB	High	55 (67.1)	101 (56.1)	69 (72.6)	0.06
	Intermediate	15 (18.3)	42 (23.3)	17 (17.9)	
	Low	12 (14.6)	37 (20.6)	9 (9.5)	
Fried score**		**2 (1–2)**	**1 (0.5-3)**	**1 (0–2)**	**0.001**
Fried status	Non-frail	**17 (20.7)**	**45 (27)**	**43 (45.3)**	**0.002**
	Pre-frail	**46 (56.1)**	**88 (48.9)**	**38 (40.0)**	
	Frail	**19 (23.2)**	**47 (26.1)**	**14 (14.7)**	
Concerns about falling, yes		**22 (26.8)**	**80 (44.4)**	**26 (27.4)**	**0.003**

The data are presented as *n* (%) or *mean ± SD or **median (p25-p75)

Bold = *p*<0.05

## Discussion

In this study, we observed a significant difference over time based on the level of declared care plan implementation on the SPPB score, walking speed, Fried status, and concerns about falling, in the group that partially implemented the care plan which seems to stand out negatively in comparison to both not implemented and fully. We did not find a significant difference in fall history over the past three months among participants identified as being at risk of losing functional independence and reduced intrinsic capacity, between their entry into the prevention program and one year after the proposal of a personalized care plan guided by comprehensive assessment.

Despite the proposal of a personalized care plan related to identified fall risk factors, no significant difference was observed in the participant’s fall history during the past three months between their entry into the prevention program and one year after the proposal of a personalized care plan. The adaptation of the care plan, which varies for each individual based on the risks identified during the initial in-depth assessment, constitutes a “multifactorial intervention.” This type of intervention is globally recommended for fall prevention in older adults [25] and has been shown to be effective in reducing fall rates in several meta-analyses, although there is heterogeneity among the studies [26–28]. Several factors may contribute to the lack of reduction in the number of fallers in our study : adherence to the care plan was low, with only a minority of participants reporting full implementation, making it difficult to expect the optimal benefit. However, there was no significant difference in fall history at the second in-depth assessment based on the level of care plan implementation. All participants received at least recommendations for healthy aging, and it has been suggested by a meta-analysis that while multifactorial interventions reduce fall rates compared to usual care, this difference disappears when compared to usual care accompanied by general fall prevention advices [28]. Following the comprehensive ICOPE assessment, the care plan may have recommended physical exercise and environmental adaptations based on the identified need, two essential elements in fall prevention [29, 30]. Unlike some interventional studies that have demonstrated a reduction in fall rates, our program did not include a specialist specifically dedicated to the implementation of these recommendations [31, 32]. It has been suggested that there is no significant reduction in fall rates when necessary interventions primarily refer participants to other services, in contrast to studies where the intervention was actively conducted by the research team [33, 34]. In the ICOPE program, as applied in our study, many of the recommendations can only refer participants to other providers. Therefore, despite the proposal of a personalized care plan suggesting evidence-based interventions, it remains uncertain whether these interventions were actually delivered at an adequate intensity. Meta-analyses of multifactorial intervention studies have shown a reduction in fall rates after the intervention, but no reduction in the number of individuals experiencing a fall [26, 27], with one analysis reporting a slight reduction [28]. Our study was unable to track the total number of falls experienced, capturing only whether participants were fallers or non-fallers within the last three months at the time of the in-depth assessment. Additionally, the WHO’s ICOPE program is not specifically aimed at fall prevention; it is designed to prevent the decline of intrinsic capacity. Due to this focus, participants were independent at the time of inclusion. Notably, the participants initially demonstrated good mobility parameters, as evidenced by the majority showing high performance on the SPPB, a deterioration of which is recognized as a risk marker of repeated fall [35]. The population integrated into the ICOPE program, due to their initially high physical capabilities, is therefore probably not the one that would benefit the most from a multifactorial intervention on fall incidence. Literature has shown that a multifactorial intervention does not reduce fall incidence in analyses of community-dwelling seniors with a history of falls in the past 12 months, however, subgroup analysis indicates a significant reduction in fall incidence among recurrent fallers [36].

There is an association between the participants’ functional abilities and the level of declared adherence to the personalized care plan. Analysis of mobility parameters during the second in-depth assessment reveals significantly different results based on the level of care plan implementation (not implemented, partially, fully), including the SPPB score, walking speed, Fried status, and concerns about falling. The initial hypothesis is that the care plan may have had a positive impact on these parameters for participants. Literature has demonstrated that greater intensity of participation in a multifactorial intervention leads to more substantial benefits in terms of improvements in the SPPB score and frailty criteria [37]. However, it should also be considered that the level of care plan implementation may depend on the participants’ initial capabilities. Better physical or cognitive abilities are predictive of higher adherence to physical exercise or cognitive activity programs, respectively [38]. Nevertheless, the intention to follow the proposed recommendations does not seem to depend on the initial functional status [39]. The analysis of these results does not indicate a linear relationship between the level of care plan implementation and mobility parameters over time, with the “partially implemented” group which seems to stand out negatively in comparison to both not implemented and fully. These results may be affected by the timing of the second in-depth assessment, which ranges from 8 to 18 months in our population. It is known that the length of follow up can influence adherence [40, 41]. We can therefore hypothesize that a later second in-depth assessment may have contributed to lower adherence and poorer results on mobility related parameters. Exploratorily, we subsequently compared participants’ capacities on these same parameters during their initial in-depth assessment, based on their later level of care plan implementation, with these results presented in Appendix 2. Significant differences were observed from the initial assessment in the SPPB score, walking speed, and frailty criteria based on the subsequent level of care plan implementation. Participants who fully implemented the care plan were found to be more robust. While this study cannot identify the barriers to implementing the care plan, it is noted that the target population of the ICOPE program, before the onset of frailty, appears to be the group most invested in the proposed recommendations, aiming to best maintain their capacities.

The WHO’s ICOPE program is identified as one of the key components of the Fall Prevention Plan in France, targeting individuals at risk of functional decline and suggesting a personalized care plan based on a comprehensive assessment. In the data examined here, no significant difference was observed, one year after the care plan proposal, in the occurrence of falls in the past three months for participants included in the program in France. This lack of difference should be interpreted with caution, as the ICOPE program is not specifically aimed at fall prevention. Participants’ functional capacities were good at inclusion, and the fall rate for each participant is not known in our study. However, it raises questions about the implementation of the care plan beyond mere suggestion, which was generally low in our population. Subgroup analyses revealed significant differences one year after the submission of the care plan concerning mobility parameters based on the level of plan implementation, highlighting the importance of this parameter. Our study does not elucidate the factors influencing adherence to the care plan but underscores the link between the implementation of the care plan and functional level. It would be beneficial to understand the determinants of its implementation. A secondary analysis suggests that those who were initially more robust appear to have fully adhered to the recommended measures, which are precisely the individuals for whom the ICOPE program is intended, even before the onset of frailty.

Our study has limitations. The collection of the event “fall in the past three months” relies on participant self-reporting, which introduces recall bias. Compared to a prospective collection, 25% [42] to 32% [43] of older adults who fell in the past three months did not report a fall when questioned retrospectively. Additionally, we do not have data on the number of falls for each participant. While the literature generally recommends a prospective 12-months follow-up for documenting fall events, the design of our study did not make such data collection feasible. Another limitation concerns the evaluation of the care plan’s implementation level. This information was self-reported by participants during the second in-depth assessment, and no intermediate follow-up data were available to accurately determine the extent to which the initially proposed care plan was actually implemented in practice. The main strength of our study is its focus on real-life application data from the deployment of the WHO ICOPE prevention program in France, and thus explores the program data as it is currently implemented in routine practice and on a large scale [44].

## Conclusion

Our study did not find significant difference in the participant’s fall history during the past three months between their entry into the prevention program and one year after the proposal of a personalized care plan. However, significant differences in SPPB score, walking speed, Fried status, and concerns about falling were noted according to participant’s self-reported level of care plan implementation. Full adherence could potentially have a beneficial effect, but those who might derive the greatest impact are declaring not fully implementing the plan. Further studies are needed to understand the barriers to applying the recommended measures of an interventional plan in this population. The deployment of the ICOPE program in France is a new and evolving phenomenon that is improving over time. Currently, the proposal of a care plan usually refers seniors to other professionals whom they must find on their own. It might be interesting to identify more medical and paramedical professionals who could be included in the ICOPE program and could assist participants in implementing their personalized interventional care plan, thereby enhancing the care pathway following the care plan suggestion.

## Supplementary Information

Supplementary Material 1: Appendix 1 : Description of the Population at the initial in-depth assessment (N=357). Appendix 2 : Comparative table of fall history and mobility-related parameters during the initial in-depth assessment, based on subsequent care plan adherence (N=357).

## Data Availability

Data availability is subject to request from the corresponding author.

## Abbreviations

ICOPE: Integrated Care for Older People
WHO: World Health Organization
IC: Intrinsic Capacity
HCP: Healthcare Professional
ADL: Activities of Daily Living
SPPB: Short Physical Performance Battery
MMSE: Mini Mental State Examination
IADL: Instrumental Activities of Daily Living
MNA: Mini Nutritional Assessment
BMI: Body Mass Index
