# Supplementary material for: Effect of the WHO ICOPE prevention program on falls and mobility functions : a retrospective study in France
Source: BMC Geriatr. 2026 Aug 11;26:1413. doi: 10.1186/s12877-026-08073-7 (PMC13757033; doi:10.1186/s12877-026-08073-7)
Supplement: Supplementary file 1 [file 12877_2026_8073_MOESM1_ESM.docx]

**Supplementary data :**

Appendix 1 : Description of the population at the initial in-depth assessment (N = 357)

| Age*, in year | | | 77.8+/-7.3 |
| --- | --- | --- | --- |
| Gender, women | | | 231 (64.7) |
| Fall history | | | 73 (20.5) |
| Number of comorbidities** | | | 2 (1–3) |
| Number of medications* | | | 4.3+/-3.1 |
| Lifestyle, alone | | | 165 (46.2) |
| Home assistance, yes | | | 157 (44.0) |
| Cognition | MMSE* | | 26.3+/-3.2 |
| Mobility | SPPB** | | 11 (8–12) |
|  | SPPB performance | Hight | 223 (62.5) |
|  |  | Intermediate | 79 (22.1) |
|  |  | Low | 55 (15.4) |
|  | Unipedal stance, normal | | 195 (54.6) |
|  | Concerns about falling, yes | | 134 (37.6) |
|  | Fried score** | | 1 (0–2) |
|  | Fried status | Non-frail | 97 (27.2) |
|  |  | Pré-frail | 182 (51.0) |
|  |  | Frail | 78 (21.8) |
|  | IADL** | | 8 (7–8) |
| Nutrition | MNA* | | 25.9+/-3.0 |
|  | Nutritional status according to MNA | Normal | 250 (70.0) |
|  |  | At risk | 53 (14.9) |
|  |  | Poor | 3 (0.8) |
|  |  | MD | 51 (14.3) |
|  | BMI in kg/m²* | | 26.3+/-4.6 |
| The data are presented as *mean ± SD or **median (p25-p75) or n (%)  MMSE: Mini-Mental State Examination (from 0 to 30), SPPB : Short Physical Performance Battery (from 0 to 12), IADL : Instrumental Activity of Daily Living Score of Lawton (from 0 to 8), MNA : Mini Nutritional Assessment (from 0 to 30), BMI : Body Mass Index, MD : Missing Data | | | |

Appendix 2 : Comparative table of fall history and mobility-related parameters during the initial in-depth assessment, based on subsequent care plan adherence (N = 357).
